# Ecophysiological Plasticity and Bacteriome Shift in the Seagrass *Halophila stipulacea* along a Depth Gradient in the Northern Red Sea

**DOI:** 10.3389/fpls.2016.02015

**Published:** 2017-01-05

**Authors:** Alice Rotini, Astrid Y. Mejia, Rodrigo Costa, Luciana Migliore, Gidon Winters

**Affiliations:** ^1^Department of Biology, Tor Vergata UniversityRome, Italy; ^2^Department of Bioengineering (iBB), Instituto Superior Técnico, Universidade de LisboaLisbon, Portugal; ^3^The Dead Sea-Arava Science CenterNeve Zohar, Israel

**Keywords:** seagrass holobiont, plant morphometry, total phenols, photosynthetic pigments, plant–microbe interaction, marine bacteria, Gulf of Aqaba

## Abstract

*Halophila stipulacea* is a small tropical seagrass species. It is the dominant seagrass species in the Gulf of Aqaba (GoA; northern Red Sea), where it grows in both shallow and deep environments (1–50 m depth). Native to the Red Sea, Persian Gulf, and Indian Ocean, this species has invaded the Mediterranean and has recently established itself in the Caribbean Sea. Due to its invasive nature, there is growing interest to understand this species’ capacity to adapt to new conditions, which might be attributed to its ability to thrive in a broad range of ecological niches. In this study, a multidisciplinary approach was used to depict variations in morphology, biochemistry (pigment and phenol content) and epiphytic bacterial communities along a depth gradient (4–28 m) in the GoA. Along this gradient, *H. stipulacea* increased leaf area and pigment contents (Chlorophyll *a* and *b*, total Carotenoids), while total phenol contents were mostly uniform. *H. stipulacea* displayed a well conserved core bacteriome, as assessed by 454-pyrosequencing of 16S rRNA gene reads amplified from metagenomic DNA. The core bacteriome aboveground (leaves) and belowground (roots and rhizomes), was composed of more than 100 Operational Taxonomic Units (OTUs) representing 63 and 52% of the total community in each plant compartment, respectively, with a high incidence of the classes *Alphaproteobacteria*, *Gammaproteobacteria*, and *Deltaproteobacteria* across all depths. Above and belowground communities were different and showed higher within-depth variability at the intermediate depths (9 and 18 m) than at the edges. Plant parts showed a clear influence in shaping the communities while depth showed a greater influence on the belowground communities. Overall, results highlighted a different ecological status of *H. stipulacea* at the edges of the gradient (4–28 m), where plants showed not only marked differences in morphology and biochemistry, but also the most distinct associated bacterial consortium. We demonstrated the pivotal role of morphology, biochemistry (pigment and phenol content), and epiphytic bacterial communities in helping plants to cope with environmental and ecological variations. The plant/holobiont capability to persist and adapt to environmental changes probably has an important role in its ecological resilience and invasiveness.

## Introduction

The small tropical seagrass *Halophila stipulacea* (Forsk) Ascherson is considered native to the Red Sea, Persian Gulf, and Indian Ocean (Den Hartog, 1970; Lipkin, 1975; El Shaffai, 2011). It is the most dominant seagrass species in the northernmost Gulf of Aqaba (GoA), forming discontinuous meadows (Angel et al., 1995; Al-Rousan et al., 2011; El Shaffai, 2011; Sharon et al., 2011; Mejia et al., 2016) in both shallow and deep environments (1–50 m depth; Sharon et al., 2009, 2011; Winters et al., 2016), and constituting an integral component of the coral reef ecosystem (El Shaffai, 2011; Winters et al., 2016). This seagrass is a putative Lessepsian migrant to the Mediterrian Sea, and over the last 150 years it has become established in many parts of the Mediterranean Sea (reviewed by Gambi et al., 2009; Sghaier et al., 2011). *H. stipulacea* is also rapidly spreading in the Caribbean Sea (Willette et al., 2014), where it is already displacing native seagrasses and associated communities (Steiner and Willette, 2015). The potential threat to local biodiversity posed by this species is considered serious: *H. stipulacea* is included in the “100 Worst Invasive Alien Species in the Mediterranean” (Streftaris and Zenetos, 2006). With the recent increase in capacity of the Suez Canal (July 2015), and with the Mediterranean Sea being one of the regions warming fastest under climate change (Marbà and Duarte, 2010), there is great concern about favoring the invasive character of *H. stipulacea* in the Mediterranean, which could have adverse implications on the slow growing and endemic *P. oceanica* meadows.

The invasiveness of *H. stipulacea* could be promoted by several plastic traits, which allow this species to thrive in a broad range of conditions, including different salinity, light and water temperatures (Por, 1971, reviewed by Gambi et al., 2009). *H. stipulacea* is able to modify its morphology in response to changes in environmental conditions: it shows increasing leaf size along depth gradients (Hulings, 1979; Lipkin, 1979; Schwarz and Hellblom, 2002), small leaf size (width and length) under high light levels (Schwarz and Hellblom, 2002), and/or high temperature and hydrodynamics (Procaccini et al., 1999; Mejia et al., 2016). Adaptations include also adjustments in photosynthetic responses (Schwarz and Hellblom, 2002; Sharon et al., 2009, 2011; Mejia et al., 2016) and in the synthesis of secondary metabolites, such as total phenols (Mejia et al., 2016). Clearly, gaining knowledge on the ecological and physiological features of this species in its native range, will contribute to understanding its invasive potential.

Microbial communities and their seagrass host (the ‘seagrass holobiont’, *sensu* Rosenberg et al., 2007), may have complex interactions which are mutually beneficial, as it has been observed in other host marine organisms such as corals (Meron et al., 2011), algae (Campbell et al., 2015a), and sponges (Webster et al., 2008). Microbial communities, due to their capability to rapidly shift in structure and function as a response to environmental change, may influence the health of their hosts (Zilber-Rosenberg and Rosenberg, 2008) by several means such as providing protection against pathogens (Rosenberg et al., 2007; Taylor et al., 2007), nitrogen fixation (Singh et al., 2004), and securing nutrient availability (Rout et al., 2013; Coats and Rumpho, 2014). In the same way, host condition (Marzinelli et al., 2015), rather than just environmental variables (Campbell et al., 2015a,b), may determine the structure of the closely associated microbial communities. Simultaneous assessments of seagrass ecophysiology and microbial community structure are therefore important to understand the capacity of the holobiont to persist under changing environmental conditions.

Recently, we found that *H. stipulacea* plants from different meadows, but growing at the same depth (9 m), displayed great plasticity at small spatial scales (3–6 km). Morphological and biochemical changes, as well as differences in their associated epiphytic microbiome, were found in response to the local environmental constraints (Mejia et al., 2016). However, the seagrass-microbe relationship are still poorly understood, and the extent to which the ecological status of the seagrass holobiont is affected by depth remains to be elucidated.

The aim of this study was to determine whether variations in morphology, biochemistry (pigment and phenol content) and epiphytic microbial communities of *H. stipulacea* plants growing along a 4–28 m depth gradient in the GoA occur, to assess the ecophysiological plasticity of the holobiont. Results from this multidisciplinary approach can further our understanding about the mechanisms used by *H. stipulacea* to maintain its ecological resilience and invasive capability under different environmental conditions, in both its native range and newly colonized areas.

## Materials and Methods

### Study Site

The study was conducted in a monospecific meadow of *H. stipulacea*, located on the western shores of the GoA, Northern Red Sea, Israel (29.497664°N, 34.912737°E). At this site, the shoreline is almost pristine, with very little coastal infrastructure or anthropic pressure (Mejia et al., 2016). This *H. stipulacea* meadow is considered healthy and harbors a rich and diverse community of corals (Winters et al., 2016). During the study (October 2013), the meadow had an area of about 61,900 m^2^ commencing at 4–5 m and extending to depths greater than 50 m (Sharon et al., 2011; Winters et al., 2016).

### Seagrass Sampling

**FIGURE 1:**
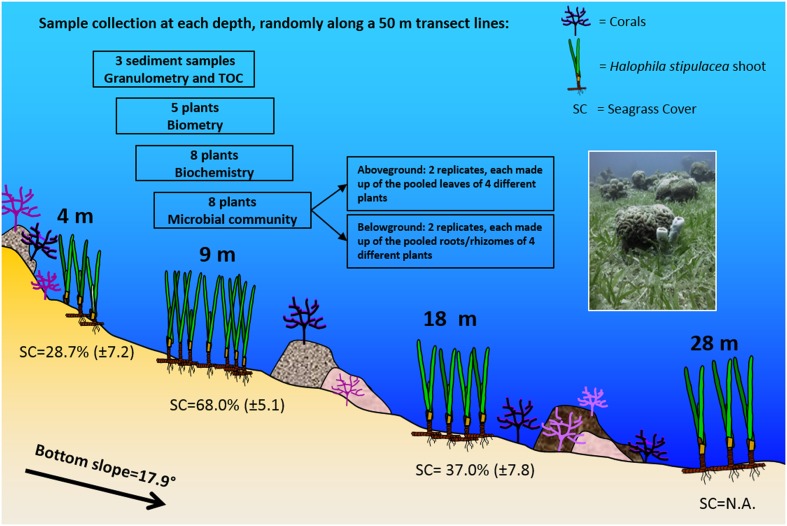
**Schematic representation of the study site and the sampling design (Eilat, Northern Red Sea).** *Halophila stipulacea* meadow showing sampling depths, bottom slope, coral occurrence, leaf biometry, and seagrass percentage cover (mean ± SD, *n* = 7–13; NA = not available), according to observations and measurements carried out in the study site during the sampling activities. The sampling design is also reported, with details of the sample collection at each depth. The photo (taken by Gidon Winters) shows the mixing of the meadow with the coral reef at the sampling site.

Sampling took place in October 2013, with average sea surface water temperature of 24°C and salinity of 40.70 PSU (NMP, 2014). Sampling was conducted using SCUBA along 50 m transect lines placed, parallel to the shore, at 4, 9, 18, and 28 m (see **Figure 1**). The sampling depths, selected based on recent mapping of the meadow (Winters et al., 2016), cover the different parts of the meadow, as follows: at 4 m, near the coral reef and the shallow edge of the meadow, at 9 m along the flat part of the sea bottom, before the drop to deeper waters, at 18 m, along the actual slope of the meadow, and at 28 m around the area where the slope straightens out again. To characterize the environment, three sediment samples were collected at random sites along the transect line with minicorers (3.0 cm diameter and 50 ml volume) and used for grain size (granulometry) measurements and Total Organic Carbon (%TOC) content analyses. The Photosynthetic Active Radiation (PAR) in the water column was measured at noon, from 0 to 28 m, using the 2π-quantum sensor of a diving-pulse amplitude modulated (PAM) fluorometer (Walz, Germany), with 3–4 light measurements made every meter. The diffuse attenuation coefficient Kd (PAR) was then calculated based on measurements made at 1 and 28 m.

At each depth, samples were taken at three sites, randomly selected, within a 5 m range, at the beginning, middle and end of the transect line. Intact plants (with rhizomes and roots) were collected at each site, separately for morphometry (five plants), biochemical analyses (eight plants), and microbial community analyses (eight plants; **Figure 1**). For the latter, the eight plants were pooled to obtain two replicates, separating underwater aboveground (leaves) and belowground (rhizomes and roots) compartments. Each replicate of the aboveground compartment was composed of at least 32 leaves, and the belowground of about 16 cm of rhizomes with roots. Four plants per replicate were pooled to obtain a representative measure of bacteriome composition at the ecosystem level, while still permitting assessment of within- and between-depth bacteriome variability through the inspection of two independent replicates per depth below- and aboveground. All samples for morphometry, biochemical and microbial community analyses were then transported to the lab and kept cold (<10°C), in the shade until further processing (within 2 h).

### Environmental Variables

To determine granulometric composition, sediment samples were pretreated to remove organic matter with 20 ml of 15% hydrogen peroxide (H_2_O_2_) and stirred for 15 min; the solution was rinsed out once bubbling ceased. The samples were then freeze dried for 72 h. Particle size distribution in the 0.02–2000 μm range was measured using a Beckman Coulter LS 13 320 Laser diffraction particle size analyzer. Sediment particles >2000 μm were weighted separately using an analytical scale. Granulometric composition was evaluated by calculating the percent frequency of seven class sizes: <63, 63–125, 125–250, 250–500, 500–1000, 1000–2000, and >2000 μm.

Sediment samples were also used for determining the %TOC in the sediments. This was measured using a Skalar Primacs SLC TOC Analyser (Skalar Analytical BV, Netherlands). For this, dry samples were grounded and split into two fractions. The first was burned at 1050°C for total carbon measurement and the second was titrated with 20% phosphoric acid to detect the inorganic carbon fraction. The subtraction yielded the %TOC.

### Plant Descriptors

Leaf morphometrics were measured using ImageJ software (version 1.47; Abramoff et al., 2004). Leaves from each depth, were scanned (Canon Lide 110 scanner) and images were used to measure leaf length (mm), width (mm) and area (mm^2^); at least 10 leaves from each plant were used for these morphometric measurements. Total phenols were extracted from the leaves (200 mg fresh weight) and rhizomes (200 mg fresh weight) of each plant in duplicate to increase confidence and quantified according to Migliore et al. (2007). Photosynthetic pigments (Chlorophyll *a* and Chlorophyll *b*, total Carotenoids) were extracted in duplicate from leaves (250 mg) of each plant, according to Wellburn (1994), modified by Rotini et al. (2013). Both phenols and photosynthetic pigment contents are expressed as mg/g of fresh weight (FW).

### Bacterial Communities

Microbial sample processing in the laboratory and sequencing data analyses followed the procedures reported in Mejia et al. (2016). Microbial cell pellets were retrieved separately from leaves and rhizomes-roots collected at each depth, followed by the extraction of microbial metagenomic DNA with the Power Soil^®^ DNA isolation kit (Mo Bio, Carlsbad, CA, USA) according to the manufacturer’s instructions. Pure DNA extracts were sent to Molecular Research LP^1^ (MR DNA, Shallowater, TX, USA; Dowd et al., 2008) for 16S rRNA gene PCR amplification and 454-pyrosequencing, using Roche GS FLX Titanium technology. The universal primers Com1 (forward, 5′-CAGCAGCCGCGGTAATAC-3′) and Com2 (reverse, 5′-CCGTCAATTCCTTTGAGTTT-3′) were used for the subsequent PCR amplification of bacterial-specific fragments of 407 bp, encompassing the V4 and V5 regions of the 16S rRNA gene, which are highly variable phylogenetically (Schmalenberger et al., 2000). The raw 454-pyrosequencing sequences were processed with the open source software MOTHUR^2^ (accessed: August 2015), according to the 454 Standard Operating Procedure (SOP) pipeline (Schloss et al., 2009). Within MOTHUR, sequences were depleted of their barcodes, primer sequences and homopolymers, and those <200 bp and with ambiguous base calls were removed. Sequences were then dereplicated, aligned against the Greengenes core-set template alignment, screened and filtered to make them overlap in the same region. Putative chimeras were identified with the “uchime” algorithm and removed. Operational Taxonomic Units (OTUs) were defined by clustering at 3% divergence (97% similarity), using the average neighbor-clustering algorithm. A final matrix (or “complete dataset”) of the OTUs abundance by sample and their taxonomic identification was built in MOTHUR and used in downstream analyses. Before OTU picking, sequences diverging by one or two nucleotides only from a more abundant sequence were considered as if they were representative reads of the most abundant sequence. The most abundant read within each OTU was then chosen and used as representative OTU sequence for taxonomic assignment. OTU representative sequences were classified with naïve Bayesian classifier in MOTHUR using the RDP taxonomy reference database-trainset 10_082014. “Unknown” sequences and those identified as “Mitochondria” were removed, while chloroplast reads were kept to enable further insights into microalgal diversity in the samples. To standardize differences in sampling effort among samples, the complete OTUs dataset was normalized by random subsampling to the common depth of 2,764 sequences (hereafter called “normalized dataset”), which was the lowest number of sequences produced by the samples in the study; this allows for adequate comparisons at community level. The complete set of raw sequences obtained in this study was deposited in GenBank at the Sequence Read Archive (SRA) under the BioProject no. PRJNA317018.

### Bacterial Community Analyses

Bacterial community analyses were performed in MOTHUR. Rarefaction curves were first built using the complete dataset to review the overall depth of sequencing of the study. Then each sample library was reduced to 5528 sequences (and a second rarefaction curve was built) to assess the bacterial richness coverage of the microenvironments analyzed. Comparative, alpha and beta diversity analyses were undertaken using the normalized dataset (2,764 sequence reads per sample) as follows: (i) Shannon index (H’) was calculated to estimate bacterial diversity; (ii) Venn diagrams were built to reveal the specific and shared bacterial OTUs by sample type, after merging the OTU profiles of the replicate samples; and iii) stacked bar plots were built to visualize bacterial community composition at high taxonomic ranks. The SIMilarity PERcentage (SIMPER) test was used to identify the contribution of the different taxa to the dissimilarity, using Bray-Curtis distances, in bacterial composition between samples. Analyses used Hellinger-transformed OTU data (square root of OTU relative abundances).

### Statistical Analysis

For environmental variables, multivariate non-parametric analysis of similarities (ANOSIM) on Euclidean distances, treating the size classes as variables, was used to test whether significant differences in granulometric composition among depths occurred. Differences in sediment %TOC and water column PAR were evaluated using ANOVA and *post hoc* Tukey’s pairwise tests.

For plant descriptors, significant differences in the mean leaf surface area among depths were evaluated using Kruskal–Wallis and Mann–Whitney pairwise tests, since data did not satisfy the homoscedasticity assumptions (Levene’s test). Differences in photosynthetic pigments and total phenols were evaluated using ANOVA and *post hoc* Tukey’s pairwise tests.

For bacterial community diversity, the non-parametric test Mann-Whitney was used to evaluate pairwise differences between depths. The normalized samples vs. OTUs contingency table and corresponding metadata were used as input for multivariate ordination analysis with the software package Canoco for Windows 4.5 (Microcomputer Power, Ithaca, NY, USA), following the procedures of Costa et al. (2006) and Keller-Costa et al. (2014). To determine the species distribution model (linear or unimodal) that fits best to our data. Detrending Correspondence Analysis (DCA) was employed to assess the extent of variation (“lengths of gradient” as implemented in Canoco) within the OTU dataset. A linear model was considered the best fit, and thus unconstrained ordination via Principal Components Analysis (PCA), was used to explore possible correlations between bacterial community structures, plant ecophysiological descriptors and environmental variables. Analyses used Hellinger-transformed OTU data (square root of OTU relative abundances).

Pearson’s correlation coefficient was used to test for relationships between environmental variables and plant descriptors using log-transformed data, and also with bacterial communities (square root transformed data). Statistical significance was defined at *p* < 0.05 (**Supplementary Table S1**).

## Results

### Environmental Variables

The granulometric composition of the sediments changed significantly along the depth gradient (ANOSIM, *R* = 0.53, *p* < 0.01; see **Supplementary Figure S1**), from gravel/coral rubble at 4 and 9 m (>40% of sediment grains sizes >2000 μm) to coarse sand at 18 and 28 m (>50% of sediment grains between sizes 250 and 2000 μm). The %TOC showed significant differences among depths (ANOVA, *F* = 9.155, *p* < 0.01). Significantly higher values were found at 9 m (0.48%) and 18 m (0.51%), compared to 4 m (0.10%) and 28 m (0.14%) (*post hoc* Tukey’s test, *p* < 0.05, see **Supplementary Figure S1**).

The photosynthetic active radiation (PAR) at noon, decreased significantly with depth (ANOVA, 259 *F* = 681.5, *p* < 0.01 and *post hoc* Tukey tests, *p* < 0.001). PAR was 182.3 ± 8.3, 258 87.7 ± 3.5, 38.7 ± 3.5, and 16.7 ± 1.2 μmol photon m^-2^ s^-1^ at 4, 9, 18, and 28 m depth, respectively.

### Plant Descriptors

The mean leaf surface area, width, and length (**Table 1**, Leaf morphometrics) showed significant differences among depths. Mean leaf area increased significantly with depth (Kruskal–Wallis, *H* = 39.14, *p* < 0.01) and showed a significant negative correlation with PAR (*R* = –0.751, *p* < 0.01).

**Table 1:** Leaf morphometrics of *Halophila stipulacea* plants along a depth gradient.

	Leaf morphometrics		
	
Depth (m)	Leaf surface area (mm^2^)	Leaf width (mm)	Leaf length (mm)
4	150.8 ± 48.7*^a^*	7.7 ± 1.7*^a^*	29.9 ± 6.6*^a^*
9	168.0 ± 56.5*^b^*	7.9 ± 1.5*^a^*	34.8 ± 7.4*^b^*
18	186.4 ± 59.1*^c^*	8.0 ± 1.6*^a^*	35.7 ± 7.8*^b^*
28	232.9 ± 49.3*^d^*	8.2 ± 1.2*^a^*	40.6 ± 6.0*^c^*


*Leaf morphometry values (means ± SD, *n* = 5) labeled with different letters (a–d) denote significant differences among depths, based on Mann–Whitney pairwise test.*

**FIGURE 2:**
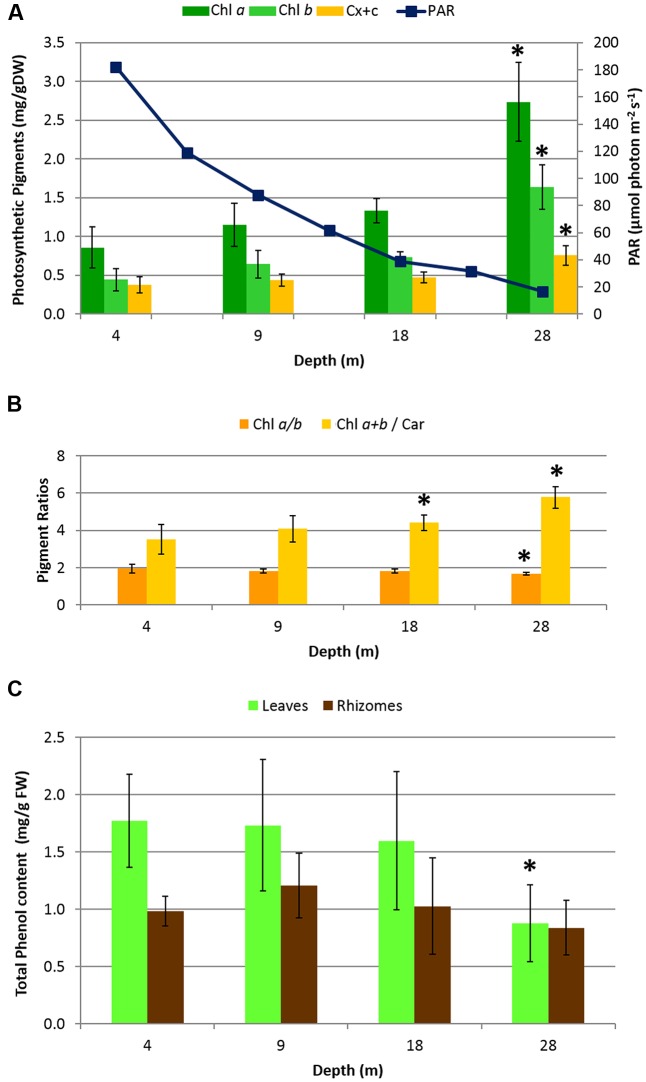
**Biochemical descriptors in *H. stipulacea* plants collected along a depth gradient.** **(A)** Mean photosynthetic pigment content in leaves (Chl *a = C*hlorophyll *a*; Chl *b = C*hlorophyll *b*; Car = total Carotenoids) and PAR measurements (taken along the 4–28 depth gradient, at noon, using the 2π-quantum sensor of the Diving-PAM fluorometer). **(B)** Pigment ratio values. **(C)** Mean total phenol content in leaves and rhizomes. Error bars represent standard deviations (*n* = 8). Significant differences among depths based on *post hoc* Tukey’s test are indicated with asterisks.

Photosynthetic pigment content (**Figure 2A**) increased with depth, as did the ratio of Chl *_a+b_*/Car, while the ratio Chl *a/b* decreased (**Figure 2B**). Significant differences along the depth gradient were found in pigments and in the ratios Chl *_a+b_*/Car and Chl *a/b* (ANOVA, *p* < 0.001) in particular, between 28 m and all the other depths (*post hoc* Tukey tests, *p* < 0.001). Photosynthetic pigment contents and ratios showed significant correlations with PAR, granulometry and plant descriptors (See **Supplementary Table S1**). The mean total phenol content (**Figure 2C**) showed significant differences only in leaves at 28 m where the lowest value was found (*post hoc* Tukey test, *p* < 0.001), while rhizomes did not show significant differences along the entire depth gradient.

### Bacterial Communities

The raw output of the 454-pyrosequencing resulted in 107,782 bacterial 16S rRNA gene sequences, comprising all samples. The processing of sequences with the MOTHUR software resulted in 84,892 sequences, assigned to a total of 6,793 OTUs, including those occurring only once or twice. On average, each individual sample was represented by 5,306 sequences, ranging from 2,764 to 9,001 sequences. The rarefaction curves analysis applied to the complete dataset (see **Supplementary Figure S2A**) was useful to demonstrate that an asymptote in OTU richness was not fully reached, even for those microenvironments characterized with a high sequencing effort (e.g., aboveground compartments at 4 m, and belowground compartment 28 m). Sectioning each curve at a common sequence depth of 5528 gene reads per sample type (see **Supplementary Figure S2B**), showed that in both leaves and rhizomes, communities sampled at 9 m were the least rich. Within each depth, belowground communities displayed, overall, higher bacterial richness values than the corresponding aboveground communities.

At most depths, the Shannon diversity index (H′) values, using the normalized dataset, were slightly lower aboveground than belowground (**Table 2**). The highest diversity values in both above- and belowground plant parts were observed at 4 m. Diversity values aboveground fluctuated among depths but belowground diversity decreased with depth. Diversity indices between 4 and 28 m were significantly different (Mann–Whitney, *p* < 0.05).

**Table 2:** Shannon diversity index of the bacterial communities associated with *H. stipulacea* along a depth gradient.

Depth (m)	Shannon diversity (H′)	
	
	Aboveground	Belowground
4	5.04 ± 0.15	5.30 ± 0.46
9	4.37 ± 0.25	4.85 ± 0.55
18	4.98 ± 0.33	5.05 ± 0.37
28	4.67 ± 0.07	4.51 ± 0.56


*Values represent the means (±SD) of two replicates per depth. The normalized dataset was used.*

**FIGURE 3:**
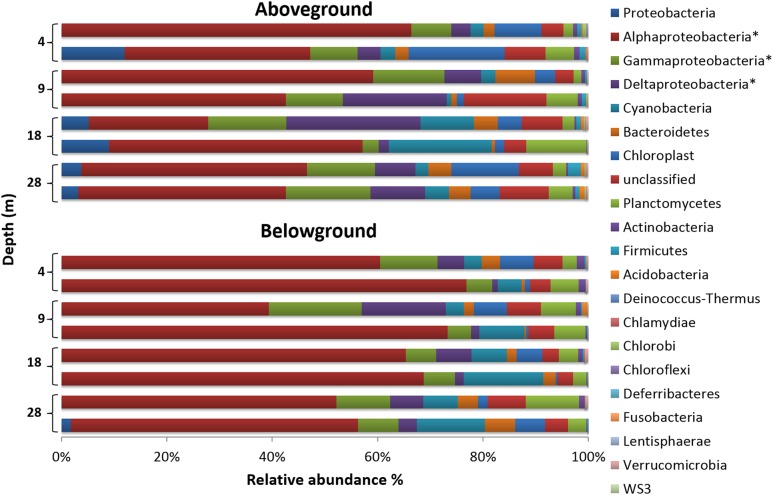
**Phylum-level composition of bacterial communities associated with *H. stipulacea* along a depth gradient.** For a better representation of the phylum Proteobacteria, its three most abundant classes (^∗^) are also shown. Bar charts display compositional data of two replicate samples per depth and plant compartment, using the normalized dataset.

*Halophila stipulacea*-associated bacterial communities (below and aboveground) were dominated by the phylum *Proteobacteria* (**Figure 3**), which accounted for 72% of the analyzed sequence reads across all samples. Within this phylum, the class *Alphaproteobacteria* was the most dominant, representing 52% of the whole community composition in the study, being more abundant aboveground (60%) than belowground (45%). Within this class, the order *Rhodobacterales* (71%) and family *Rhodobacteraceae* (71%) were the most abundant. The second and third most abundant classes were *Gammaproteobacteria* (10%) and *Deltaproteobacteria* (8%), and both were more abundant belowground than aboveground. Surprisingly, only 12% of the *Gammaproteobacteria* reads could be assigned to up to seven formally recognized orders, with *Alteromonadales* (8%) being the most abundant, while the remainder of the reads were considered unclassifiable or possessing uncertain placement at the order level. The *Deltaproteobacteria* community was mainly composed of bacteria belonging to the orders *Myxoccocales* (33%) and *Desulfobacterales* (50%), out of seven orders observed. The latter comprised the family *Desulfobulbaceae* (47%), which was the most abundant among the 12 families observed, as well as the genus *Desulfopila* (27%), the most abundant of the 32 genera observed. Overall, the 16 phyla detected comprised about 29 classes, 40 orders, 82 families, and over 100 genera. Bacteria not classifiable at the phylum level and chloroplast-derived sequences accounted for 5.2 and 6%, respectively, of the whole community composition.

The SIMPER test (see **Supplementary Table S2**) showed dissimilarities of 20.91% in the bacterial community structure between plant parts. Community dissimilarities across depths were slightly lower aboveground (range of 15–19%) than belowground (range of 19–24%). The top five taxa contributing to dissimilarities were Rhodobacteraceae (734 OTUs, 16722 seqs), Gammaproteobacteria (711 OTUs, 4240 seqs), Desulfobulbaceae (145 OTUs, 1579 seqs), Roseibium (33 OTUs, 2792 seqs), and Desulfopila (14 OTUs, 896 seqs). The combined sequences of the groups mentioned above accounted for about 60% of the normalized dataset.

**FIGURE 4:**
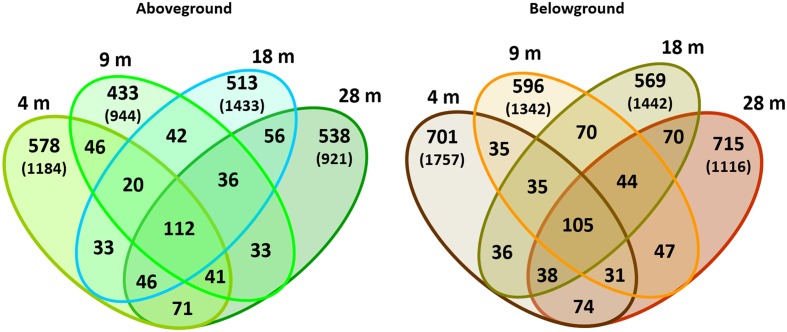
**Venn diagrams of bacterial OTUs associated with *H. stipulacea* by depth.** OTUs (specific and shared) associated with aboveground and belowground plant parts at 4, 9, 18, 28 m. Values represent two replicates per depth, using the normalized dataset. In brackets: total number of OTUs found in each plant compartment per depth using the normalized dataset.

The distribution of specific and shared OTUs showed differences by plant part and depth (**Figure 4**). In general, the total number of OTUs observed aboveground was lower than belowground (2,598 vs. 3,166 OTUs, respectively; using the normalized dataset). Bacterial communities showed a higher number of specific than shared OTUs. The shared OTUs revealed the ‘core bacteriome’ of *H. stipulacea* by plant part along the gradient (see **Supplementary Table S3**). These OTUs usually displayed high abundance values, within the range of 100–1000 sequences per OTU and represented 63 and 52% of the total number of sequences observed above and belowground, respectively. The most abundant phylotype found in the core bacteriome, belonged to the phylum *Proteobacteria*, class *Alphaproteobacteria*.

### Ordination Analysis (PCA): Environmental Variables, Plant Descriptors, and Bacterial Communities

**FIGURE 5:**
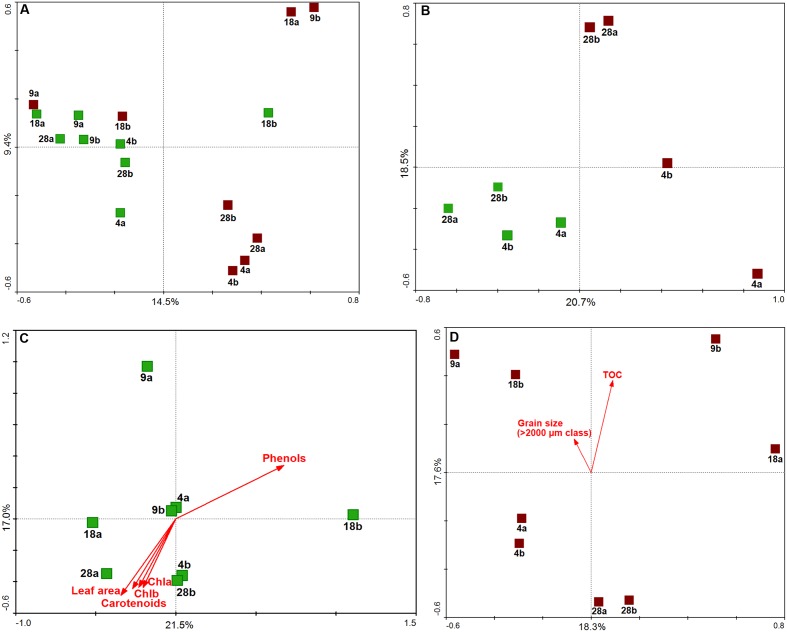
**Multivariate analysis of *H. stipulacea*-associated bacterial communities along a depth gradient (4–28 m).** Principal Component Analysis (PCA) results performed on Hellinger-transformed data using scaling on sample distances are shown as: **(A)** ordination of all samples, **(B)** ordination of only 4 and 28 m samples, **(C)** ordination of aboveground samples, **(D)** ordination of belowground samples. Aboveground samples are in green, belowground samples in brown; sample labels refer to the depths (4, 9, 18, 28 m) and replication units **(a,b)**. Samples were positioned in the ordination diagram according to their community dissimilarity, based on Euclidean distances, calculated from OTU data. In the ordination diagrams, values of plant descriptors **(C)** and environmental variables **(D)** are represented by arrows, displayed passively in the ordination space (i.e., not influencing the position of the samples). The ordering of samples, according to increasing values of each descriptor/variable, can be approximated by perpendicularly projecting sample points onto the respective arrows. The percent of explained variance for each principal componente, corresponding to the amount of variation in OTU data, is reported.

Ordination diagrams obtained by Principal Components Analysis (PCA, **Figure 5**) showed the different forces shaping the bacterial communities along the gradient. Overall, aboveground and belowground samples displayed a clearer and stronger separation by plant part than depth (**Figure 5A**). The communities at the edges of the meadows, 4 and 28 m, depicted best the simultaneous influence of parts and depths (**Figure 5B**), with the latter factor exerting greater influence on the structure of belowground communities than on aboveground communities. Both aboveground (**Figure 5C**) and belowground (**Figure 5D**) communities showed higher within-depth variability at 9 and 18 m than at 4 and 28 m, where replicate samples (within each depth) were clearly more similar to each other. The PCA diagrams illustrated the grouping of aboveground communities from 28 m which had the largest leaves, highest carotenoids and chlorophyll contents, and lowest phenols (**Figure 5C**); and of belowground communities with lower TOC values and smaller grain sizes.

## Discussion

A multidisciplinary approach was used to investigate the ecophysiological status of a seagrass holobiont (*sensu* Rosenberg et al., 2007), along a depth gradient (4–28 m) in the GoA (Northern Red Sea). Differences in some environmental characteristics, like seagrass cover and presence of corals were observed along the gradient (**Figure 1**). The lowest plant percent cover of *H. stipulacea* was observed at 4 m where corals were present, while the highest percent cover was found at 9 m where corals were absent. The shallowest edge of the gradient (4 m) likely had also the highest hydrodynamics, as suggested by predominantly large sediment composition of gravel and coral rubble (>2000 μm) and the lowest %TOC (**Supplementary Figure S1**). Although high water clarity was observed throughout most of the water column in the meadow (Kd = 0.115), at the deepest site (28 m) the available PAR reduced to about 5% of the surface PAR. Low levels of PAR can limit growth in seagrasses (e.g., Duarte, 1991), but maybe not so much in *H. stipulacea* which grows deeper than 40 m in the study site (Sharon et al., 2011; Winters et al., 2016). *H. stipulacea* plants showed clear morphological and biochemical variation, in response to changing environmental conditions along the depth gradient. The significant increase of photosynthetic pigments and leaf surface area with depth, as well as the significant changes in the Chl *_a+b_*/Car and Chl *a/b* ratios and their correlation with PAR, illustrates the mechanism of the physiological acclimation of *H. stipulacea* to low light. The fine-tuning of the photosynthetic surface and pigment content in response to PAR in *H. stipulacea* was also observed by Lipkin (1979); Schwarz and Hellblom (2002), and Mejia et al. (2016) under changing light regimes. This study further demonstrates that photoacclimation capability is an important adaptation to growing in low light conditions.

Total phenol content is considered a suitable indicator of the ecophysiological status of seagrasses (Migliore et al., 2007; Rotini et al., 2013), since seagrasses are known to modulate their phenol content in response to stressful environmental conditions (e.g., Rotini et al., 2011; Darnell and Heck, 2013; Silva et al., 2013; Arnold et al., 2014). The reduced phenol concentrations at 28 m may be related to a simultaneous reduction in the phenolic biosynthesis and increase of phenolic mobilization, both triggered by low light regimes. Indeed, changing light regimes are known to influence phenolic biosynthesis (Vergeer et al., 1995; Rotini et al., 2013) and patterns of storage to sustain seagrasses growth (Alcoverro et al., 2001; Collier et al., 2009). Plants at 28 m were exposed to the lowest irradiance of the gradient and it is possible that the phenolic biosynthesis is reduced, while the mobilization of carbon-based storage products, like phenolics, is promoted to maintain metabolic processes under reduced light conditions. Similarly, Mejia et al. (2016) observed increased concentrations of total phenols in *H. stipulacea* tissues under high light regimes. Further studies are needed to explore the complexity of the role and dynamics of secondary metabolites in highly plastic seagrass species such as *Halophila* sp.

The epiphytic bacterial community also varied with environmental conditions, with shifts in community structures across depths and belowground communities at 28 m the most different to the others. Nevertheless, for each plant part, a conserved core community composition (“core bacteriome”) occurred at all depths. In general, the bacterial community belowground was more diverse (H’) than aboveground (**Table 2**). The high bacterial diversity observed at 4 m in both plant parts may be related to stronger hydrodynamics occurring at this depth. Increased hydrodynamic regimes cause a coarse granulometry in the sediments and the re-suspension of fine particles and nutrients in the water column. Increased availability and re-suspension of nutrients in the water column (García-Martínez et al., 2009; Guevara et al., 2014) and sediments (Uku et al., 2007) along the shoreline can open several micro-niches for different bacteria to thrive in, resulting in higher bacterial diversity.

The dominant-core bacteriome composition of *H. stipulacea*, encompassed many different phylotypes many of which were found both on both above and belowground biomass. Interestingly, we found a similar core bacteriome composition to the one reported by Mejia et al. (2016) from different meadows at the same study area and by Weidner et al. (2000) in the GoA, both on *H. stipulacea*. Along with a large core bacteriome across all aboveground samples (63% of the total bacterial 16S rRNA gene reads retrieved from leaves), high variability was observed in the epiphytic bacteriome structure of *H. stipulacea* leaves. The bacterial taxonomic assembly may follow an event-driven pattern as suggested elsewhere for seaweeds (Burke et al., 2011a,b; Campbell et al., 2015a), resulting from the low probability that first colonizers of emerging leaves will be exactly the same for different leaf onset events. In support of this hypothesis, we found the highest bacterial diversity at 4 m aboveground where the increased hydrodynamic regimes, including tide currents, could drive more variable planktonic communities at the local scale. Higher bacterial community variability, at a given depth, was found in both above and belowground samples from 9 and 18 m, suggesting an important role of environmental fluctuations in eroding stable bacterial community assembly. We consider it likely that mature communities will carry out similar metabolic functions despite taxonomic differences, as observed for other symbiotic assemblages (Burke et al., 2011a; Fan et al., 2012). Host-driven deterministic forces and casual microbial recruitment may simultaneously influence bacteriome structuring on *H. stipulacea*. Therefore, beside the influence of early colonizers and abiotic oscillations, plants may play a role in the preservation of a symbiotic consortium of bacteria, which helps to secure fundamental plant metabolic functions (Burke et al., 2011a). The widespread abundance and dominance of the class *Alphaproteobacteria* on *H. stipulacea* along the gradient and at other meadows within the GoA (Weidner et al., 2000; Mejia et al., 2016) may be due to its natural abundance in the water column and its capability to thrive in multiple environmental conditions, especially in oligotrophic coastal waters (Dang et al., 2008; Witt et al., 2012; Elifantz et al., 2013). In particular, the family *Rhodobacteraceae* has been significantly linked with variations in water column salinity, oxygen saturation, pH, nitrate concentration, and temperature (Buchan et al., 2005; Campbell et al., 2015b). In sediments, multiple factors can influence the bacterial community structure of seagrass rhizomes and roots. These include the quality and quantity of root exudates available to belowground tissues (Smith et al., 2004; García-Martínez et al., 2005; Cúcio et al., 2016), the initial composition of the sediment microbiota (Hahn, 2003; Jensen et al., 2007), and the roots architecture (Küsel et al., 1999; García-Martínez et al., 2005).

Overall, our results suggest a changing ecological status of the *H. stipulacea* holobiont along a depth gradient (4–28 m). There were not only marked differences in plant morphology and biochemistry, but also the associated bacterial consortium. The plant and microbial community responses to the changes in environmental conditions are likely to be contributors to the health of the holobiont along the gradient. The structure and diversity of the bacterial communities associated with *H. stipulacea* suggest a possible functional relationship between seagrass and microbes, in the framework of the “holobiont theory” proposed for corals (Rosenberg et al., 2007; Zilber-Rosenberg and Rosenberg, 2008). Plant–microbe interactions may be also important to invasive plants and may aid colonization and expansion in new territories. Particularly, the rhizosphere-associated communities are known to persist attached to the roots-rhizomes in new environments (Coats and Rumpho, 2014; Cúcio et al., 2016). Our findings suggest an important role for morphology, biochemistry (pigment and phenol content) and epiphytic bacterial communities in increasing plant tolerance for environmental and ecological variations and thus improving their ecological resilience and invasiveness capacity. We recommend a multidisciplinary approach to further explore the influence of the microbiome on seagrass holobiont health. Shotgun metagenomic approaches could determine the functional diversity of the associated microbial communities; while variations in the proteome (Migliore et al., 2007; Dattolo et al., 2013) and metabolome (Hasler-Sheetal and Holmer, 2015) could investigate the influence of environmental gradients in shaping the seagrass holobiont. These approaches will improve the effectiveness of monitoring programs, allowing a more comprehensive assessment of the health status of the seagrass holobiont.

## Author Contributions

GW and LM conceived and designed the study. GW designed the sampling strategy; GW, AM, and AR performed the sampling, contributed to the preparation of the samples and the analyses of plant biometry. AR performed biochemical analyses and statistical analysis of plant descriptors and environmental variables. AM performed microbial samples preparation, DNA extractions and community analyses. RC performed the community level statistical analyses. AM and AR wrote the manuscript; all authors contributed to the discussion and approved the final manuscript.

## Conflict of Interest Statement

The authors declare that the research was conducted in the absence of any commercial or financial relationships that could be construed as a potential conflict of interest.

## Footnotes

1 http://www.mrdnalab.com

2 http://www.mothur.org/
