# Supplementary material for: Ecophysiological Plasticity and Bacteriome Shift in the Seagrass Halophila stipulacea along a Depth Gradient in the Northern Red Sea
Source: Front Plant Sci. 2017 Jan 5;7:2015. doi: 10.3389/fpls.2016.02015 (PMC5221695; doi:10.3389/fpls.2016.02015)
Supplement: Supplementary file 1 [file Data_Sheet_1.docx]

***Supplementary Material***

**Ecophysiological plasticity and bacteriome shift in the seagrass *Halophyla stipulacea* along a depth gradient in the Northern Red Sea**

**Alice Rotini, Astrid Y. Mejia, Rodrigo Costa , Luciana Migliore, Gidon Winters***

*** Correspondence:** Gidon Winters, wintersg@adssc.org

**Supplementary Figures and Tables**

**
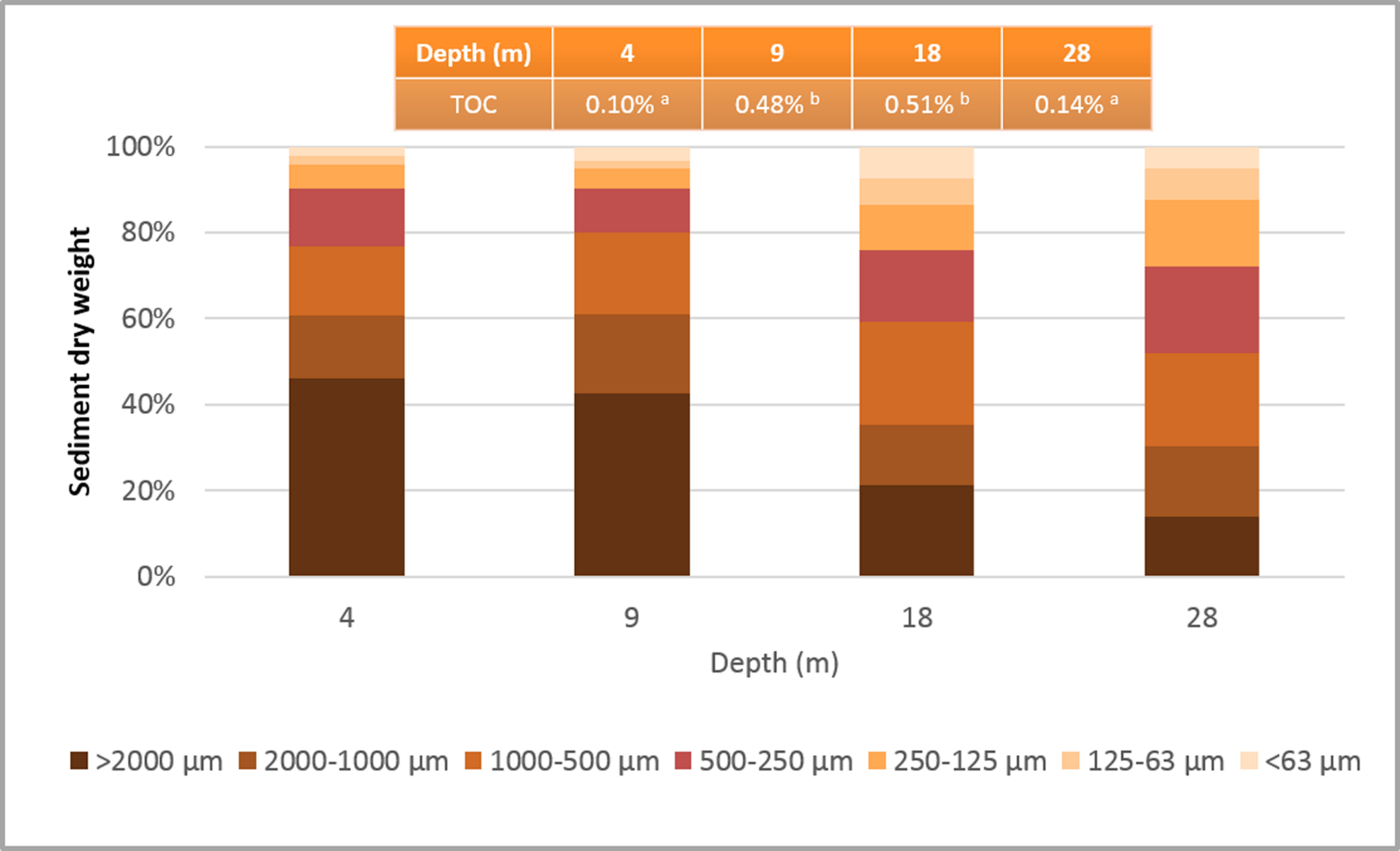
**

**Figure S1.** Granulometric composition and %TOC of sediments in the *Halophila stipulacea* meadow along a depth gradient. Granulometric composition of sediments is expressed as a percentage of dry weight of seven granulometric size classes and represent means of three measurements. %TOC is reported in table on top of the bars, values represent means of three measurements and statistical significance is shown (letters a - b): TOC values labeled with the same letter did not differ significantly based on post-hoc Tukey’s test.

**
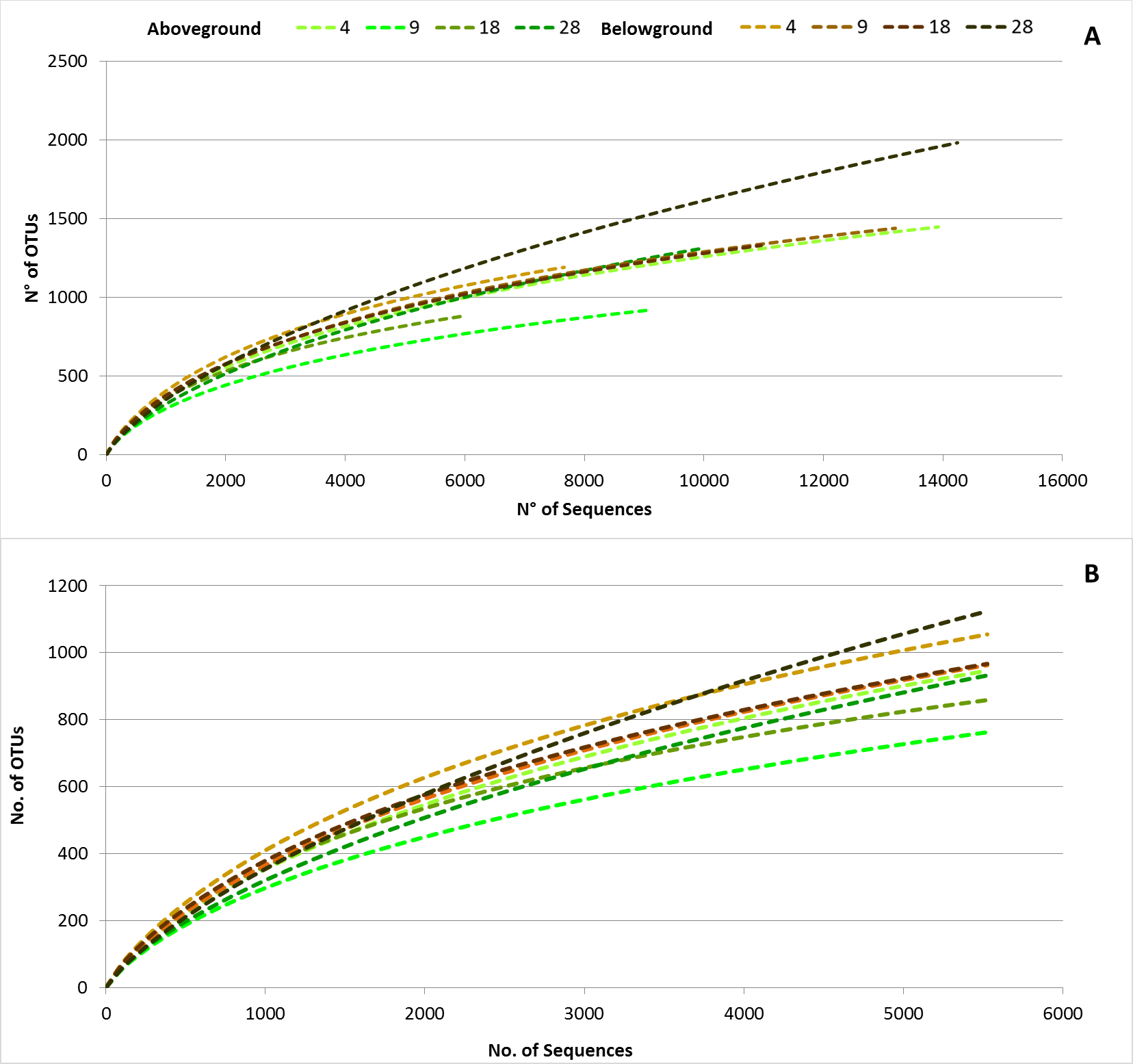
**

**Figure S2.** Rarefaction curves of the bacterial communities associated with *Halophila stipulacea* along a depth gradient, built using: A) the complete dataset; B) a common sequencing depth of 5528 sequences per sample. Values represent the mean of two replicates per depth.

**Table S1.** Pearson’s correlation coefficient values obtained from the analysis of environmental variables, biochemical descriptors and bacterial communities associated with *Halophila stipulacea* plants along a depth gradient in the Gulf of Aqaba, Red Sea, Israel. Statistical significance shown by p<0.001=***; p<0.01=**; p<0.05=*.

| **Pearson’s correlation coefficient results (PAST v. 3.1)** | | |
| --- | --- | --- |
| ***Variables*** | ***R value*** | ***P significant*** |
| Granulometry (>2000) and PAR | R = 0.90097 | *** |
| Granulometry (>2000) and Leaf Area | R= -0.754 | ** |
| Granulometry (>2000) and Phenols (leaves) | R= 0.716 | ** |
| Granulometry (>2000) and Chl tot | R= -0.830 | *** |
| Granulometry (>2000) and Car | R= -0.830 | *** |
| PAR and Leaf Area | R= -0.75077 | ** |
| PAR and Phenols (leaves) | R= 0.72272 | ** |
| PAR and Chl tot | R = -0.90197 | *** |
| PAR and Car | R= -0.8633 | *** |
| Leaf Area and Chl tot | R = 0.684 | * |
| Leaf Area and Car | R = 0.686 | * |
| Phenols (leaves) and Chl tot | R = -0.804 | ** |
| Phenols (leaves) and Car | R= -0.81696 | ** |
| Chl tot and Car | R= 0.9901 | *** |
| Bacterial community aboveground |  | |
| Shannon diversity and *Alphaproteobacteria* abundance | R= -0.7495 | * |
| *Alphaproteobacteria* and *Gammaproteobacteria* abundance | R= -0.94125 | *** |
| *Alphaproteobacteria* and *Deltaproteobacteria* abundance | R= -0.91068 | *** |
| *Gammaproteobacteria* and *Deltaproteobacteria* abundance | R= 0.88079 | *** |
| *Alphaproteobacteria* and *Acidobacteria* abundance | R= -0.90309 | *** |
| *Deltaproteobacteria* and *Acidobacteria* abundance | R= 0.97428 | *** |
| Bacterial community belowground |  | |
| Shannon diversity and *Alphaproteobacteria* abundance | R=-0.7419 | * |
| Shannon diversity and *Gammaproteobacteria* abundance | R=0.73062 | * |
| Shannon diversity and *Unclassified* abundance | R=0.73062 | * |
| Shannon diversity and *Deltaproteobacteria* abundance | R=0.71947 | * |
| *Alphaproteobacteria* and *Deltaproteobacteria* abundance | R=-0.7815 | * |
| *Planctomycetacia* and *Flavobacteria* abundance | R=-0.73761 | * |
| *Cyanobacteria* and *Actinobacteria* abundance | R=-0.76317 | * |

**Table S2**. SIMilarity PERcentage (SIMPER) test analysis, showing the percent contribution to the dissimilarity of the bacterial communities per depth. The 10 top taxa and their individual contribution to the total dissimilarity between groups are shown.

| ***SIMPER ANALYSIS – Bacterial communities*** | | | | | |  | | | |  |
| --- | --- | --- | --- | --- | --- | --- | --- | --- | --- | --- |
| ***Aboveground*** | | | | | |  | | | |  |
| Depths | | *4 vs. 9* | | | *4 vs. 18* | | | | |  |
|  | | Total % dissimilarity | | 15.93% | Total % dissimilarity | 18.93% | | | |  |
| No. | | Taxon | | % contribution | Taxon | % contribution | | | |  |
| 1 | | Rhodobacteraceae | | 5.31 | Desulfopila | 5.61 | | | |  |
| 2 | | Roseibium | | 4.73 | Rhodobacteraceae | 5.21 | | | |  |
| 3 | | Flavobacteriaceae | | 4.24 | Alphaproteobacteria | 4.60 | | | |  |
| 4 | | Myxococcales | | 4.18 | Gammaproteobacteria | 3.92 | | | |  |
| 5 | | Planctomycetaceae | | 3.88 | Sphingobacteriales | 3.58 | | | |  |
| 6 | | Desulfopila | | 3.63 | Desulfobulbaceae | 3.37 | | | |  |
| 7 | | Blastopirellula | | 3.49 | Planctomycetaceae | 3.18 | | | |  |
| 8 | | Zeaxanthinibacter | | 2.84 | Muricauda | 3.07 | | | |  |
| 9 | | Erythrobacter | | 2.56 | Flavobacteriaceae | 2.91 | | | |  |
| 10 | | Deltaproteobacteria | | 2.21 | Hoeflea | 2.28 | | | |  |
| Depths | | *4 vs. 28* | | | *9 vs. 18* | | | | |  |
|  | | Total % dissimilarity | | 16.46% | Total % dissimilarity | 19.09% | | | |  |
| No. | | Taxon | | % contribution | Taxon | % contribution | | | |  |
| 1 | | Rhodobacteraceae | | 4.94 | Rhodobacteraceae | 5.79 | | | |  |
| 2 | | Alphaproteobacteria | | 4.86 | Desulfopila | 5.64 | | | |  |
| 3 | | Desulfopila | | 3.74 | Alphaproteobacteria | 4.77 | | | |  |
| 4 | | Planctomycetaceae | | 3.73 | Gammaproteobacteria | 4.30 | | | |  |
| 5 | | Myxococcales | | 3.45 | Desulfobulbaceae | 4.06 | | | |  |
| 6 | | Rhodopirellula | | 3.05 | Roseibium | 4.03 | | | |  |
| 7 | | Flavobacteriaceae | | 2.84 | Myxococcales | 3.54 | | | |  |
| 8 | | Gammaproteobacteria | | 2.77 | Blastopirellula | 3.20 | | | |  |
| 9 | | Acidomicrobiales | | 2.54 | Sphingobacteriales | 3.11 | | | |  |
| 10 | | Zeaxanthinibacter | | 2.45 | Hoeflea | 3.10 | | | |  |
| Depths | | *9 vs. 28* | | | *18 vs. 28* | | | | |  |
|  | | Total % dissimilarity | | 14.77% | Total % dissimilarity | 17.64% | | | |  |
| No. | | Taxon | | % contribution | Taxon | % contribution | | | |  |
| 1 | | Myxococcales | | 5.96 | Rhodobacteraceae | 6.24 | | | |  |
| 2 | | Roseibium | | 5.79 | Desulfopila | 6.08 | | | |  |
| 3 | | Alphaproteobacteria | | 5.17 | Alphaproteobacteria | 5.10 | | | |  |
| 4 | | Acidomicrobiales | | 3.96 | Gammaproteobacteria | 4.41 | | | |  |
| 5 | | Gammaproteobacteria | | 3.95 | Desulfobulbaceae | 3.90 | | | |  |
| 6 | | Planctomycetaceae | | 2.78 | Myxococcales | 3.71 | | | |  |
| 7 | | Flammeovirgaceae | | 2.60 | Sphingobacteriales | 3.63 | | | |  |
| 8 | | Sphingobacteriales | | 2.51 | Hoeflea | 2.80 | | | |  |
| 9 | | Deltaproteobacteria | | 2.49 | Blastopirellula | 2.57 | | | |  |
| 10 | | Erythrobacter | | 2.31 | Arcobacter | 2.48 | | | |  |
| ***Belowground*** | | | | | | | | | |  |
| Depths | | *4 vs. 9* | | | | *4 vs. 18* | | | | |
|  | | Total % dissimilarity | | 21.79% | | Total % dissimilarity | | 21.26% | | |
| No. | | Taxon | | % contribution | | Taxon | | % contribution | | |
| 1 | | Rhodobacteraceae | | 4.94 | | Desulfobulbaceae | | 5.29 | | |
| 2 | | Desulfopila | | 3.68 | | Desulfopila | | 5.08 | | |
| 3 | | Sphingobacteriales | | 3.62 | | Gammaproteobacteria | | 4.46 | | |
| 4 | | Desulfobulbaceae | | 3.00 | | Rhodobacteraceae | | 4.42 | | |
| 5 | | Clostridiales | | 2.92 | | Alphaproteobacteria | | 3.31 | | |
| 6 | | Cohaesibacter | | 2.84 | | Planctomycetaceae | | 3.27 | | |
| 7 | | Blastopirellula | | 2.79 | | Rhodopirellula | | 3.12 | | |
| 8 | | Roseibium | | 2.75 | | Clostridiales | | 2.87 | | |
| 9 | | Muricauda | | 2.61 | | Zeaxanthinibacter | | 2.53 | | |
| 10 | | Desulfovibrio | | 2.57 | | Flammeovirgaceae | | 2.49 | | |
| Depths | | *4 vs. 28* | | | | *9 vs. 18* | | | | |
|  | | Total % dissimilarity | | 19.20% | | Total % dissimilarity | | | 23.86% | |
| No. | | Taxon | | % contribution | | Taxon | | | % contribution | |
| 1 | | Roseibium | | 15.01 | | Rhodobacteraceae | | | 5.60 | |
| 2 | | Rhodobacteraceae | | 4.83 | | Desulfopila | | | 4.79 | |
| 3 | | Gammaproteobacteria | | 3.93 | | Desulfobulbaceae | | | 4.76 | |
| 4 | | Myxococcales | | 3.75 | | Gammaproteobacteria | | | 3.44 | |
| 5 | | Desulfobulbaceae | | 2.94 | | Sphingobacteriales | | | 3.35 | |
| 6 | | Desulfosarcina | | 2.36 | | Planctomycetaceae | | | 3.19 | |
| 7 | | Sphingomonadales | | 2.25 | | Alphaproteobacteria | | | 2.95 | |
| 8 | | Flammeovirgaceae | | 2.09 | | Rhodopirellula | | | 2.85 | |
| 9 | | Zeaxanthinibacter | | 2.09 | | Pelagibius | | | 2.67 | |
| 10 | | Blastopirellula | | 1.79 | | Blastopirellula | | | 2.62 | |
| Depths | | *9 vs. 28* | | | | *18 vs. 28* | | | | |
|  | | Total % dissimilarity | | 22.13% | | Total % dissimilarity | | | 22.92% | |
| No. | | Taxon | | % contribution | | Taxon | | | % contribution | |
| 1 | | Roseibium | | 13.16 | | Roseibium | | | 11.55 | |
| 2 | | Desulfobulbaceae | | 4.97 | | Rhodobacteraceae | | | 5.28 | |
| 3 | | Desulfopila | | 4.90 | | Desulfopila | | | 3.54 | |
| 4 | | Rhodobacteraceae | | 4.48 | | Sphingobacteriales | | | 3.44 | |
| 5 | | Gammaproteobacteria | | 3.57 | | Desulfobulbaceae | | | 2.69 | |
| 6 | | Alphaproteobacteria | | 3.20 | | Blastopirellula | | | 2.66 | |
| 7 | | Rhodopirellula | | 3.08 | | Cohaesibacter | | | 2.65 | |
| 8 | | Planctomycetaceae | | 3.04 | | Myxococcales | | | 2.62 | |
| 9 | | Acidomicrobiales | | 2.19 | | Desulfovibrio | | | 2.43 | |
| 10 | | Clostridiales | | 2.06 | | Clostridiales | | | 2.05 | |

**Table S3.** The core microbiome (using the normalized dataset) associated with the plant compartments of *Halophila stipulacea* along a depth gradient. OTU = Number of Operational Taxonomic Units; Seq = Number of sequences observed.

| **Aboveground** | | | | | | | | | | | | | | |
| --- | --- | --- | --- | --- | --- | --- | --- | --- | --- | --- | --- | --- | --- | --- |
| Phylum | OTU | Seqs | Class | OTU | Seqs | Order | OTU | Seqs | Family | OTU | Seqs | Genus | OTU | Seqs |
| *Actinobacteria* | 2 | 92 | *Actinobacteria* | 2 | 92 | *Acidimicrobiales* | 2 | 92 | *Unclassified* | 2 | 92 | *Unclassified* | 2 | 92 |
| *Bacteroidetes* | 3 | 156 | *Flavobacteria* | 3 | 156 | *Flavobacteriales* | 3 | 156 | *Flavobacteriaceae* | 3 | 156 | *Muricauda* | 1 | 50 |
|  |  |  |  |  |  |  |  |  |  |  |  | *Zeaxanthinibacter* | 1 | 71 |
|  |  |  |  |  |  |  |  |  |  |  |  | *Unclassified* | 1 | 35 |
| *Planctomycetes* | 11 | 438 | *Planctomycetacia* | 11 | 438 | *Planctomycetales* | 11 | 438 | *Planctomycetaceae* | 11 | 438 | *Blastopirellula* | 3 | 57 |
|  |  |  |  |  |  |  |  |  |  |  |  | *Rhodopirellula* | 2 | 130 |
|  |  |  |  |  |  |  |  |  |  |  |  | *Unclassified* | 6 | 251 |
| *Proteobacteria* | 76 | 11500 | *Alphaproteobacteria* | 63 | 10324 | *Alphaproteobacteria*  *Incertae sedis* | 2 | 52 | *Alphaproteobacteria*  *Incertae sedis* | 2 | 52 | *Geminicoccus* | 2 | 52 |
|  |  |  |  |  |  | *Rhodobacterales* | 34 | 8210 | *Rhodobacteraceae* | 34 | 8210 | *Loktanella* | 1 | 103 |
|  |  |  |  |  |  |  |  |  |  |  |  | *Roseibium* | 2 | 948 |
|  |  |  |  |  |  |  |  |  |  |  |  | *Unclassified* | 31 | 7159 |
|  |  |  |  |  |  | *Rhodospirillales* | 4 | 181 | *Rhodospirillaceae* | 4 | 181 | *Pelagibius* | 1 | 61 |
|  |  |  |  |  |  |  |  |  |  |  |  | *Unclassified* | 3 | 120 |
|  |  |  |  |  |  | *Rhizobiales* | 7 | 448 | *Cohaesibacteraceae* | 1 | 28 | *Cohaesibacter* | 1 | 28 |
|  |  |  |  |  |  |  |  |  | *Hyphomicrobiaceae* | 2 | 28 | *Unclassified* | 2 | 28 |
|  |  |  |  |  |  |  |  |  | *Phyllobacteriaceae* | 2 | 324 | *Hoeflea* | 1 | 270 |
|  |  |  |  |  |  |  |  |  |  |  |  | *Unclassified* | 1 | 54 |
|  |  |  |  |  |  |  |  |  | *Rhodobiaceae* | 1 | 23 | *Anderseniella* | 1 | 23 |
|  |  |  |  |  |  |  |  |  | *Unclassified* | 1 | 45 | *Unclassified* | 1 | 45 |
|  |  |  |  |  |  | *Sphingomonadales* | 1 | 62 | *Erythrobacteraceae* | 1 | 62 | *Erythrobacter* | 1 | 62 |
|  |  |  |  |  |  | *Unclassified* | 15 | 1371 | *Unclassified* | 15 | 1371 | *Unclassified* | 15 | 1371 |
|  |  |  | *Deltaproteob* | 3 | 711 | *Desulfobacterales* | 2 | 342 | *Desulfobulbaceae* | 2 | 342 | *Desulfopila* | 1 | 323 |
|  |  |  |  |  |  |  |  |  |  |  |  | *Unclassified* | 1 | 19 |
|  |  |  |  |  |  | *Myxococcales* | 1 | 369 | *Unclassified* | 1 | 369 | *Unclassified* | 1 | 369 |
|  |  |  | *Gammaproteobacteria* | 10 | 465 | *Alteromonadales* | 1 | 7 | *Alteromonadaceae* | 1 | 7 | *Haliea* | 1 | 7 |
|  |  |  |  |  |  | *Gammaproteobacteria*  *incertae_sedis* | 1 | 25 | *Gammaproteobacteria*  *Incertae sedis* | 1 | 25 | *Unclassified* | 1 | 25 |
|  |  |  |  |  |  | *Unclassified* | 8 | 433 | *Unclassified* | 8 | 433 | *Unclassified* | 8 | 433 |
| *Cyanobacteria Chloroplast* | 16 | 1641 | *Cyanobacteria* | 10 | 985 | *Cyanobacteria incertae sedis* | 10 | 985 | *Family_II* | 6 | 894 | *GpIIa* | 6 | 894 |
|  |  |  |  |  |  |  |  |  | *Unclassified* | 4 | 91 | *Unclassified* | 4 | 91 |
|  |  |  | *Unclassified* | 1 | 17 | *Unclassified* | 1 | 17 | *Unclassified* | 1 | 17 | *Unclassified* | 1 | 17 |
|  |  |  | *Chloroplast* | 5 | 639 | *Chloroplast*  *Incertae sedis* | 5 | 639 | *Chloroplast* | 5 | 639 | *Streptophyta* | 5 | 639 |
| *Unclassified* | 4 | 68 | *Unclassified* | 4 | 68 | *Unclassified* | 4 | 68 | *Unclassified* | 4 | 68 | *Unclassified* | 4 | 68 |
| **Total number of OTUs:112, sequences: 13895.** | | | | | | | | | | | | | | |

| **Belowground** | | | | | | | | | | | | | | |
| --- | --- | --- | --- | --- | --- | --- | --- | --- | --- | --- | --- | --- | --- | --- |
| Phylum | OTU | Seqs | Class | OTU | Seqs | Order | OTU | Seqs | Family | OTU | Seqs | Genus | OTU | Seqs |
| *Actinobacteria* | 1 | 13 | *Actinobacteria* | 1 | 13 | *Acidimicrobiales* | 1 | 13 | *Unclassified* | 1 | 13 | *Unclassified* | 1 | 13 |
| *Bacteroidetes* | 9 | 364 | *Flavobacteria* | 5 | 196 | *Flavobacteriales* | 5 | 196 | *Flavobacteriaceae* | 5 | 196 | *Actibacter* | 1 | 9 |
|  |  |  |  |  |  |  |  |  |  |  |  | *Tenacibaculum* | 1 | 13 |
|  |  |  |  |  |  |  |  |  |  |  |  | *Zeaxanthinibacter* | 1 | 111 |
|  |  |  |  |  |  |  |  |  |  |  |  | *Unclassified* | 2 | 63 |
|  |  |  | *Sphingobacteria* | 4 | 168 | *Sphingobacteriales* | 4 | 168 | *Flammeovirgaceae* | 1 | 8 | *Unclassified* | 1 | 8 |
|  |  |  |  |  |  |  |  |  | *Unclassified* | 3 | 160 | *Unclassified* | 3 | 160 |
| *Chlorobi* | 1 | 40 | *Ignavibacteria* | 1 | 40 | *Ignavibacteriales* | 1 | 40 | *Ignavibacteriaceae* | 1 | 40 | *Ignavibacterium* | 1 | 40 |
| *Planctomycetes* | 10 | 398 | *Planctomycetacia* | 9 | 384 | *Planctomycetales* | 9 | 384 | *Planctomycetaceae* | 9 | 384 | *Blastopirellula* | 1 | 67 |
|  |  |  |  |  |  |  |  |  |  |  |  | *Rhodopirellula* | 2 | 100 |
|  |  |  |  |  |  |  |  |  |  |  |  | *Unclassified* | 6 | 217 |
|  |  |  | *Phycisphaerae* | 1 | 14 | *Phycisphaerales* | 1 | 14 | *Phycisphaeraceae* | 1 | 14 | *Phycisphaera* | 1 | 14 |
| *Proteobacteria* | 68 | 8961 | *Alphaproteobacteria* | 40 | 7069 | *Alphaproteobacteria incertae sedis* | 1 | 16 | *Alphaproteobacteria incertae sedis* | 1 | 16 | *Geminicoccus* | 1 | 16 |
|  |  |  |  |  |  | *Rhodobacterales* | 21 | 5875 | *Rhodobacteraceae* | 21 | 5875 | *Loktanella* | 1 | 59 |
|  |  |  |  |  |  |  |  |  |  |  |  | *Roseibium* | 1 | 1721 |
|  |  |  |  |  |  |  |  |  |  |  |  | *Unclassified* | 19 | 4095 |
|  |  |  |  |  |  |  |  |  | *Unclassified* | 1 | 14 | *Unclassified* | 1 | 14 |
|  |  |  |  |  |  | *Rhodospirillales* | 2 | 91 | *Rhodospirillaceae* | 2 | 91 | *Pelagibius* | 1 | 51 |
|  |  |  |  |  |  |  |  |  |  |  |  | *Unclassified* | 1 | 40 |
|  |  |  |  |  |  | *Rhizobiales* | 7 | 445 | *Cohaesibacteraceae* | 1 | 157 | *Cohaesibacter* | 1 | 157 |
|  |  |  |  |  |  |  |  |  | *Hyphomicrobiaceae* | 2 | 44 | *Unclassified* | 2 | 44 |
|  |  |  |  |  |  |  |  |  | *Phyllobacteriaceae* | 2 | 217 | *Hoeflea* | 1 | 181 |
|  |  |  |  |  |  |  |  |  |  |  |  | *Unclassified* | 1 | 36 |
|  |  |  |  |  |  |  |  |  | *Rhodobiaceae* | 1 | 13 | *Anderseniella* | 1 | 13 |
|  |  |  |  |  |  | *Sphingomonadales* | 1 | 31 | *Erythrobacteraceae* | 1 | 31 | *Erythrobacter* | 1 | 31 |
|  |  |  |  |  |  | *Unclassified* | 8 | 611 | *Unclassified* | 8 | 611 | *Unclassified* | 8 | 611 |
|  |  |  | *Deltaproteobacteria* | 10 | 1113 | *Desulfobacterales* | 8 | 670 | *Desulfobulbaceae* | 8 | 670 | *Desulfopila* | 3 | 552 |
|  |  |  |  |  |  |  |  |  |  |  |  | *Desulfosarcina* | 1 | 33 |
|  |  |  |  |  |  |  |  |  |  |  |  | *Unclassified* | 4 | 85 |
|  |  |  |  |  |  | *Desulfovibrionales* | 1 | 19 | *Unclassified* | 1 | 19 | *Unclassified* | 1 | 19 |
|  |  |  |  |  |  | *Myxococcales* | 1 | 424 | *Unclassified* | 1 | 424 | *Unclassified* | 1 | 424 |
|  |  |  | *Gammaproteobacteria* | 17 | 773 | *Alteromonadales* | 2 | 69 | *Alteromonadaceae* | 2 | 69 | *Haliea* | 2 | 69 |
|  |  |  |  |  |  | *Gammaproteobacteria incertae sedis* | 4 | 173 | *Gammaproteobacteria incertae sedis* | 4 | 173 | *Unclassified* | 4 | 173 |
|  |  |  |  |  |  | *Unclassified* | 11 | 531 | *Unclassified* | 11 | 531 | *Unclassified* | 11 | 531 |
|  |  |  | *Unclassified* | 1 | 6 | *Unclassified* | 1 | 6 | *Unclassified* | 1 | 6 | *Unclassified* | 1 | 6 |
| *Cyanobacteria Chloroplast* | 6 | 197 | *Cyanobacteria* | 6 | 197 | *Cyanobacteria incertae sedis* | 6 | 197 | *Family_II* | 4 | 149 | *GpIIa* | 4 | 149 |
|  |  |  |  |  |  |  |  |  | *Unclassified* | 2 | 48 | *Unclassified* | 2 | 48 |
|  | 4 | 1406 | *Chloroplast* | 4 | 1406 | *Chloroplast incertae sedis* | 4 | 1406 | *Chloroplast* | 4 | 1406 | *Bacillariophyta* | 3 | 767 |
|  |  |  |  |  |  |  |  |  |  |  |  | *Streptophyta* | 1 | 639 |
| *Unclassified* | 6 | 152 | *Unclassified* | 6 | 152 | *Unclassified* | 6 | 152 | *Unclassified* | 6 | 152 | *Unclassified* | 6 | 152 |
| **Total number of OTUs:105, sequences: 11531.** | | | | | | | | | | | | | | |
